# Molecular mechanisms of methylglyoxal-induced aortic endothelial dysfunction in human vascular endothelial cells

**DOI:** 10.1038/s41419-020-2602-1

**Published:** 2020-05-28

**Authors:** Jae Hyuk Lee, Amna Parveen, Moon Ho Do, Min Cheol Kang, Silvia Yumnam, Sun Yeou Kim

**Affiliations:** 1 College of Pharmacy, Gachon University, #191, Hambakmoero, Yeonsu-Gu, Incheon, 21936 Republic of Korea; 2 Korea Food Research Institute, 245, Nongsaengmyeong-Ro, Iseo-Myeon, Wanju_Gun, Jeollabuk-Do 55365 Republic of Korea; 3 Gachon Institute of Pharmaceutical Science, Gachon University, #191 Hambakmoe-Ro, Yeonsu-Gu, Incheon, 21936 Republic of Korea; 4 Gachon Medical Research Institute, Gil Medical Center, Inchon, 21565 Republic of Korea

**Keywords:** Macroautophagy, Macroautophagy

## Abstract

Methylglyoxal (MGO)-induced cellular apoptosis, oxidative stress, inflammation, and AGE formation are specific events that induce vascular endothelial cell (EC) toxicity in endothelial dysfunction (ED). MGO accumulates quickly in various tissues and plays a prominent role in the pathogeneses of several diabetic complications. Unbalanced angiogenesis is a gateway to the development of diabetic complications. EC apoptosis and autophagy work together to regulate angiogenesis by interacting with different angiogenic factors. In addition to understanding the deep mechanism regarding MGO-dependent autophagy/apoptosis may provide new therapeutic applications to treat diabetes and diabetic complications. Therefore, the present study aimed to investigate the regulatory effects of MGO-induced autophagy and apoptosis on angiogenesis in HAoEC and to elucidate the molecular mechanisms to discover new target base therapy for diabetes and diabetic complications. In MGO-stimulated HAoEC, protein expression was identified using a western blot, autophagosomes were observed by bio-transmission electron microscopy (TEM), and cell autophagic vacuoles and flux were measured using a confocal microscope. We found that MGO significantly induced autophagy, declined the pro-angiogenic effect, decreased proliferation, migration, and formation of tube-like structures, and increased autophagic vacuoles, flux and autophagosomes in the HAoEC in a dose-dependent manner. We observed that MGO-induced autophagic cell death and inhibited the ROS-mediated Akt/mTOR signaling pathway. MGO also triggered apoptosis by elevating the cleaved caspase-3 to Bax/Bcl-2 ratio and through activation of the ROS-mediated MAPKs (p-JNK, p-p38, and p-ERK) signaling pathway. Collectively, these findings suggest that autophagy and apoptosis inhibit angiogenesis via the ROS-mediated Akt/mTOR and MAPKs signaling pathways, respectively, when HAoEC are treated with MGO.

## Introduction

Diabetes mellitus (DM) is a major social problem^1^. A previous study reported that the prevalence of DM is on the rise, and 425 million patients live with diabetes worldwide and are expected to increase by 47% to 629 million in 2045^2^. DM, generally known as diabetes, is a type of metabolic and inflammatory disease characterized by hyperglycemia^3,4^. DM leads to hyperglycemia, which slowly leads to secondary complications including inflammation and cell death, among others (retinopathy, nephropathy, neuropathy, vasculopathy, etc.)^3^. Although the management of diabetes mainly focusses on treating hyperglycemia, there is evidence to suggest that the imbalance of vascular angiogenesis potentially contributes to the pathogeneses of diabetic complications^5^. Balancing in the angiogenesis of vascular endothelial cells (EC) is associated with vein and aortic endothelial dysfunction (ED)^6^. Several studies have suggested that EC might result in apoptosis, necrosis, and autophagy, which activate angiogenesis imbalance via interacting with various angiogenic factors^7–9^. Therefore, targeting autophagy/apoptosis in relation to other causative factors may provide new therapeutic applications to ameliorate diabetic complications.

Methylglyoxal (MGO) is the main precursor of advanced glycation end-product (AGE) production. The accumulation of MGO has been associated with the pathogenesis of diabetes, vascular complications, and several age-related chronic inflammatory diseases such as cardiovascular disease, Parkinson’s diseases, and Alzheimer’s disease^10,11^. As a highly reactive dicarbonyl metabolite, MGO can react with proteins at lysine and arginine residues, via the Maillard reaction, to form AGEs^12^. MGO can accumulate quickly in various tissues and plays a prominent role in the pathogeneses of several diabetic complications^13^. A recent report demonstrated that MGO can cause ED, microvascular complications (nephropathy, retinopathy, and neuropathy), macrovascular (atherosclerosis, heart failure, impaired revascularization, and wound healing), obesity, β-cells toxicity, insulin resistance, and age-related diseases (hypertension, cancer, and central nervous system; dementia, Parkinson’s disease, schizophrenia, and anxiety disorders)^11^. Aminoguanidine (AG) has been reported to prevent diabetic complications by preventing the AGE formation and decreasing plasma and aortic MGO levels^14–16^. Therefore, aminoguanidine was used to confirm the underline mechanism associated with MGO to treat diabetes.

Blood vessels consist of arteries, veins, or capillaries. In comparison to the wall of veins, the wall of an artery consists of three layers such as intima, media, and adventitia. Both arteries and veins contain the vasa vasorum, which has been reported to be involved in angiogenesis and in the initiation and progression of atherosclerosis^17^ and considered as a potential target for the treatment of cardiovascular disease. However, ED in arteries directly induces structural changes (atherosclerosis) and functional changes (ED) due to hypertension, dyslipidemia, diabetes, and obesity/metabolic syndromes that, in turn, induce arterial stiffness and impair vasodilation^18^. As previously reported that MGO accumulated in arterial walls and aorta of spontaneously hypertensive rats with aging and vascular contractile dysfunction in high blood pressure^19,20^. In addition, previous studies have reported that diabetic patients have a high risk of arterial diseases such as coronary heart disease, peripheral vascular disease, and carotid artery stenosis^21,22^. Therefore, it expected that the role of MGO in ECs from arteries may be more important than that of veins in diabetic complications.

Angiogenesis is generally defined as the formation of new blood vessels from existing capillaries, and it plays an important role in maintaining vascular health^23^. Angiogenesis can be dysfunctional in the peripheral vasculature, which can cause cardiac mortality due to delayed wound healing, exacerbated peripheral limb ischemia, and reduced collateral vessel development^24^. Also, angiogenesis including cell migration and proliferation are both necessary constituents for wound closure^25,26^. Recently, the main concern involves age-related alteration in normal physiological functions like diabetes. Disruption of normal wound healing process such as proliferative phase, remodeling phase in diabetes^27^. Therefore, impaired wound healing is a common impediment of diabetes that has potentially devastating consequences on suffering patients. The mechanisms underlying diabetes in relation to angiogenesis disorders are complex. However, several molecular mechanisms have been suggested, including MGO-induced vascular apoptosis in diabetic complications, mainly through mitochondrial membrane potential impairment, reactive oxygen species (ROS) production, ED, and glucotoxicity^28–30^. In a previous study, Xu et al. revealed that MGO promotes autophagy and VEGF receptor 2 (VEGFR2) in order to reduce vein endothelial angiogenesis, and described a new mechanism behind diabetic complications related to imbalanced angiogenesis in human umbilical vein EC (HUVEC)^31^. In addition, AGEs, glucose (sugars), and palmitate (lipids) trigger autophagy in vascular EC^32,33^. Previous studies have stated that autophagy and apoptosis are triggered by several common upstream signals and that they exhibit responses in a mutually exclusive routine^34^. Autophagy is not only involved in EC survival and death, but it also modulates other important EC functions including homeostasis/thrombosis, angiogenesis, and NO production^35^. Evidence has increasingly indicated that autophagy and apoptosis share major cellular mechanisms, either directly or indirectly, and that they play a potential role in regulating EC angiogenesis^36,37^. Considering all the above facts, we realized the importance of MGO-dependent autophagy/apoptosis in EC from arteries. This autophagy/apoptosis in relation to causative factors such as MGO may provide a new therapeutic approach to ameliorate diabetic diseases interlinked with aortic ED. Therefore, the current study was designed to explore deep mechanisms regarding MGO-dependent autophagy/apoptosis relation in human aortic EC to find out novel antidiabetic and antidiabetic complications therapy.

## Materials and methods

### Chemicals and reagents

Glyoxal solution (GO), MGO solution, and d-(+)-Glucose were purchased from Sigma (St. Louis, MO, USA). Bovine serum albumin (RD tech, C0082—100), Matrigel matrix (Invitrogen, MA, USA), Endothelial cell growth medium 2, and MV 2 (PromoCell, Heidelberg, Germany) were also purchased from respective companies. p38 (9212S), phospho-p38 (p-p38; 9211S), ERK1/2 (9102S), phospho-ERK1/2 (p-ERK1/2; 9101S), JNK (9252S), phospho-JNK (p-JNK; 9251S), mTOR (7C10, 2983s), phospho-mTOR (p-mTOR, Ser2448, 2971s), Akt (9272S), phospho-Akt (p-Akt; 9271S), Caspase-3 (9661S), and Alexa Fluor® 555 Phalloidin (8953S) were purchased from Cell Signaling Technology (Danvers, MA, USA). Bcl-2 (sc-492), Bax (sc-493), and VEGF-C (sc-9047) were purchased from Santa Cruz Biotechnology (Santa Cruz, CA, USA). Beclin-1 (NB500249) was purchased from Novus Biologicals (Littleton, CO, USA). Hoechst 33342 (Waltham, MA, USA) was purchased from Thermo Fisher Scientific. Cyto-ID® autophagy detection kits were obtained from Enzo Life Science (Farmingdale, NY, USA). p62 (610833) and fluorescein isothiocyanate (FITC) Annexin V apoptosis detection kit I (San Diego, CA, USA) were purchased from BD Biosciences Pharmingen.

### Cell cultures

Human aortic EC (HAoEC; C-12271), HUVEC; C-12206, and human dermal microvascular EC (HDMEC; C-12212) were purchased from PromoCell (Heidelberg, Germany). HUVEC were cultured in EGM-2 (Lot No. 406M001), and HAoEC and HDMEC were cultured in MV-2 (Lot No. 411M081), and all cell lines were supplemented and mixed with 1% penicillin/streptomycin. All cell lines were cultured at 37 °C in a humidified incubator containing 5% CO_2_.

### Cell viability and morphological examinations

Cell viability was determined using Methyl Thiazolyl Blue Tetrazolium Bromide (MTT) assays. HAoEC, HUVEC, and HDMEC were seeded in 96-well plates at 1.0 × 10^4^ and at 1.2 × 10^4^ cells/well, respectively. After incubation for 24 h, cells were treated with MGO at different concentrations (0.6, 0.8, and 1.0 mM) at two different times post-incubation (1 and 24 h). A final concentration of 0.1 mg/mL MTT solution was added to each well. The respective plates were incubated at 37 °C for 2 h in 5% CO_2_, after which the medium was carefully removed and 100 μl dimethyl sulfoxide was added to each well. The absorbance at 570 nm was measured using a microplate reader (Molecular Devices, CA, USA). The morphology of the cells was observed using the IncuCyte Zoom imaging system (Essen Bioscience, MI, USA).

### Autophagy detection assays

HAoEC, HUVEC, and HDMEC were seeded in 8-well plates at 3.0 × 10^5^ cells/well. After 24 h incubation, the cells were treated with several concentrations of MGO (0.6, 0.8, and 1 mM) for 1 h or 24 h. Cells were also treated with a mixture of 500 nM rapamycin and 10 μM chloroquine as a positive control. The Cyto-ID® autophagy detection kit (Enzo Life Sciences, Plymouth Meeting, PA) was used to measure autophagic flux, according to the manufacturer’s instructions. Samples were briefly dyed with Cyto-ID Green Dye and Hoechst 33342 at 37 °C for 30 min. The cells were then washed with 1× assay buffer and were observed under a laser scanning confocal microscope (Nikon Al+, Japan). A standard FITC filter set was used to visualize the autophagic flux. Also, the DAPI filter was used to visualize nuclear signals.

### Bio-transmission electron microscope (TEM) analysis

HAoEC were incubated with 1.0 mM MGO for 1 h. Cells were fixed in 2.5% glutaraldehyde, washed with phosphate-buffered saline (PBS), and postfixed with 1% OsO_4_ for 3 h. The samples were then washed again, dehydrated with alcohol, and embedded in propylene oxide and Epon812 media. After that, ultrathin sections were examined under a Bio‐High‐Voltage Electron Microscope (JEM‐1000BEF, at 1000 kV) (JEOL, Tokyo, Japan).

### Cell proliferation assays

Cell proliferation assays were performed using BrdU cell proliferation assay kits (Cell signaling, Danvers, USA). Briefly, HAoEC, HUVEC, and HDMEC were seeded into a 24-well plate (4.0 × 10^4^ cells/well) and were incubated for 24 h. Subsequently, cells were treated with different concentrations (0.6, 0.8, and 1.0 mM) of MGO in serum-free media, along with BrdU, and were incubated for 48 h. After incubation, the amount of BrdU-positive incorporation was determined by the ELISA method, following the manufacturer’s instructions.

### Cell migration assays

HAoEC, HUVEC, and HDMEC were seeded in 96-well plates at 4.0 × 10^4^ cells/well. After 24 h incubation, confluent monolayers of cultured cells were scratch wounded with a Wound Maker tool (Essen Bioscience, Ann Arbor, MI). The medium was removed, and cells were washed with PBS before being treated with different concentrations (0.6, 0.8, and 1.0 mM) of MGO in a serum-free medium. Following 24 h incubation, the scratch wells were observed every two hours using IncuCyte ZOOM (Essen Bioscience).

### Tube formation assays

To assess tube formation, 96-well plates were coated with 50 μL of Matrigel matrix and were incubated at 37°C to allow gel formation. After 30 min, coated wells were washed with PBS, and HAoEC, HUVEC, and HDMEC (2.0 × 10^4^) were seeded into each well with a vehicle or different concentrations (0.6, 0.8, and 1.0 mM) of MGO for 24 h. Tube formation was viewed using a microscope (Nikon, Tokyo, Japan).

### Cell apoptosis assays

Apoptosis and necrosis production was evaluated using the FITC/Annexin V Apoptosis Detection Kit I (BD Biosciences Pharmingen, San Diego, CA, USA) according to the manufacturer’s protocol. Briefly, HAoEC were seeded into a 6-well plate (3.0 × 10^5^ cells/well) were incubated for 24. After 24 h, cells were treated with 0.6, 0.8, or 1.0 mM of MGO for 1 and 24 h. Cells were washed with cold PBS and the cells were resuspended in 1× binding buffer with FITC Annexin V and propidium iodide (PI) at room temperature (25 °C) for 15 min. Then, the samples were analyzed using flow cytometry (FACSCalibur^TM^; Becton-Dickinson, San Jose, CA, USA) within 1 h.

### Intracellular ROS detection

Intracellular ROS was measured using DCF-DA as a fluorogenic dye, which interacts with hydroxyl, peroxyl, and other ROS. Briefly, 1.0 × 10^6^ cells were seeded in a 60 mm dish and were incubated overnight in a 5% CO_2_ atmosphere at 37 °C. After 24 h, HAoEC were treated with 0.6, 0.8, or 1.0 mM of MGO for 1 h. The cells were then stained with 30 μM DCF-DA. After 30 min incubation at 37 °C, cells were harvested and the change in fluorescence intensity was analyzed using a flow cytometer (FACSCalibur™; Becton-Dickinson, San Jose, CA, USA).

### Western blot analysis

HAoEC, HUVEC, and HDMEC were harvested, washed with cold Dulbecco’s PBS (1×), and lysed in a PRO-PREP™ Protein Extraction Solution (iNtRON, Seoul, Korea) containing protease and phosphatase inhibitors. The cell lysates were centrifuged at 12,000 rpm for 20 min at 4 °C. Then, the supernatants were collected, and Bradford Assays were used to quantify the proteins. Equivalent amounts of each protein were separated using sodium dodecyl sulfate polyacrylamide gel electrophoresis gels and were transferred to nitrocellulose blotting membranes using a Trans-Blot® Turbo™ Blotting System. The membranes were blocked with 5% skimmed milk in TBST for 1 h at 20 to 25 °C and were then subjected to primary antibodies overnight at 4 °C. After 24 h incubation, membranes were incubated with secondary antibodies conjugated with horseradish peroxidase for 1 h at room temperature. The protein signals were visualized using a ChemiDoc XRS + imaging system (Bio-Rad, CA, USA).

### Immunofluorescence analysis

HAoEC, HUVEC, and HDMEC treated as described were fixed with 10% formalin for 15 min at room temperature (25 °C). The cells were then washed with PBS, dyed with Alex Fluor® 555 Phalloidin to F-actin for 30 min and Hoechst 33342 for 5 min. The stained cells were mounted with mounting medium to fixation (Thermo Scientific, Waltham, MA, USA). Immunofluorescence intensity was evaluated using a NIS-Elements imaging software was used to quantify the F-actin intensity.

### Statistical analysis

The data are expressed as means ± SEM. Statistical comparisons were carried out between control and experimental groups using Bonferroni’s test for multiple comparisons of one-way analysis of variance using GraphPad Prism 5.0 (GraphPad Software Inc., San Diego, CA, USA). *P* values < 0.05 were considered to be statistically significant.

## Results

### MGO induces LC3-I/LC3-II expression in vascular ECs

**Fig. 1:**
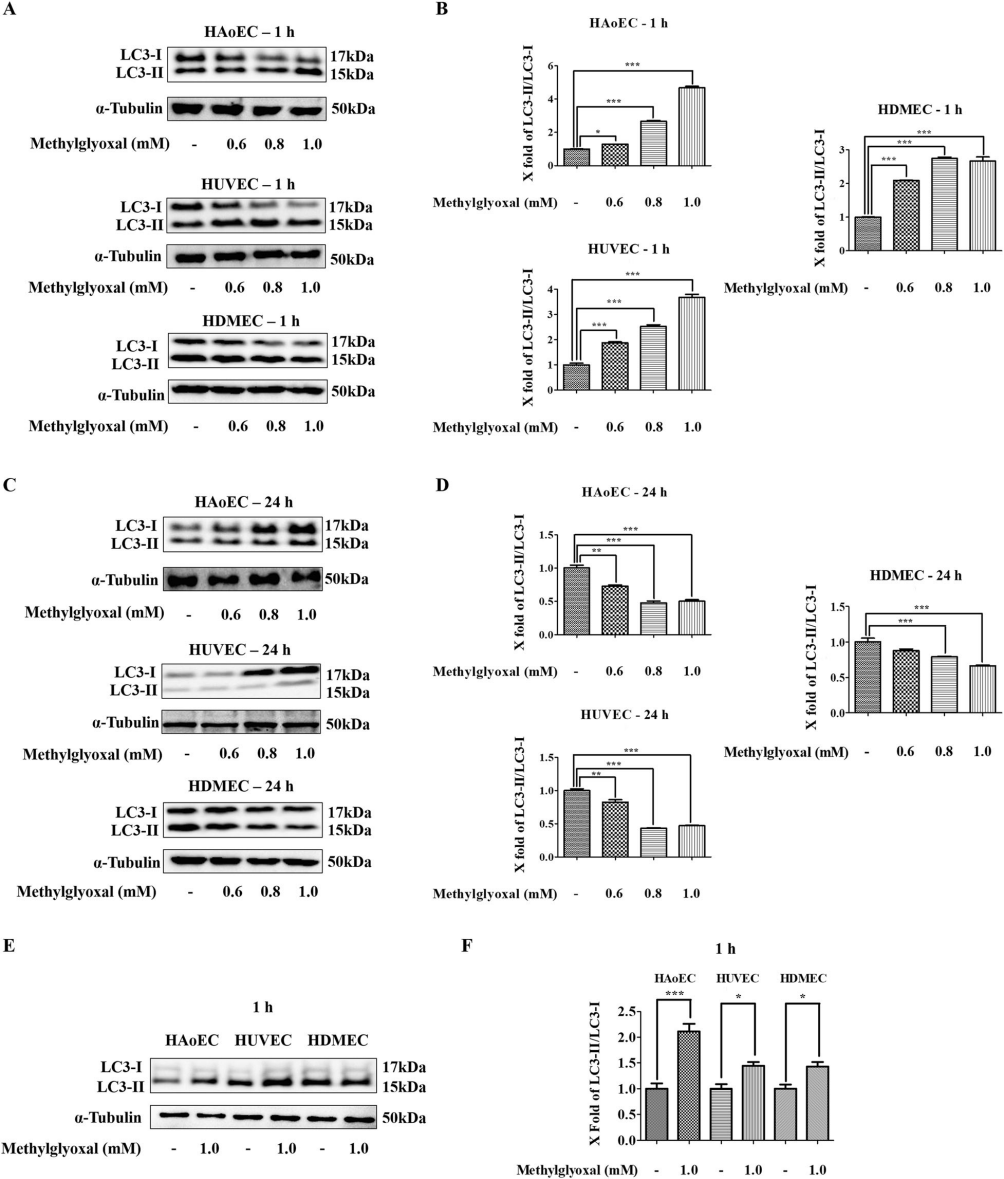
Effects of MGO-induced autophagy in vascular endothelial cells. **a**–**c**, **e** MGO-treated HAoEC, HUVEC, and HDMEC were evaluated for the expression of autophagy-associated proteins LC3-I and LC3-II. **b**–**d**, **f** The protein expression levels of LC3-I, II, and α-tubulin were analyzed by western blot at 1 h and 24 h of MGO treatment. All data are shown as means ± SEM. *N* = 3 (*p < 0.05, **p < 0.01, ***p < 0.001 vs. Control).

In this study, the effect of MGO-induced autophagy on HAoEC, HUVEC, and HDMEC was investigated. The effect of MGO on HUVEC has already been reported; however, HAoEC and HDMEC share many characteristics with HUVEC. Therefore, in this study, it was hypothesized that MGO may exert similar effects on HDMEC and HAoEC. Consequently, autophagy induction by MGO was identified by changes in the LC3-I and LC3-II autophagic marker proteins. HAoEC, HUVEC, and HDMEC were treated with several concentrations of MGO (0.6, 0.8, and 1.0 mM) for 1 and 24 h. As shown in Fig. 1, at 1 h, the autophagy-related LC3-II/LC3-I ratio increased in a dose-dependent manner (Fig. 1a, b). However, at 24 h, the autophagy-related LC3-II/LC3-I ratio decreased (Fig. 1c, d). The data clearly indicates the presence of the autophagy-related LC3-II/LC3-I ratio at 1 h suggesting MGO-induced autophagy in vascular EC at 1 h. However, the expression of LC3-II/LC3-I ratio was found to be more in HAoEC as compared to HUVEC and HDMEC (Fig. 1e, f) representing HAoEC is more susceptible to autophagy.

### MGO induces autophagic vacuoles and flux in vascular ECs

**Fig. 2:**
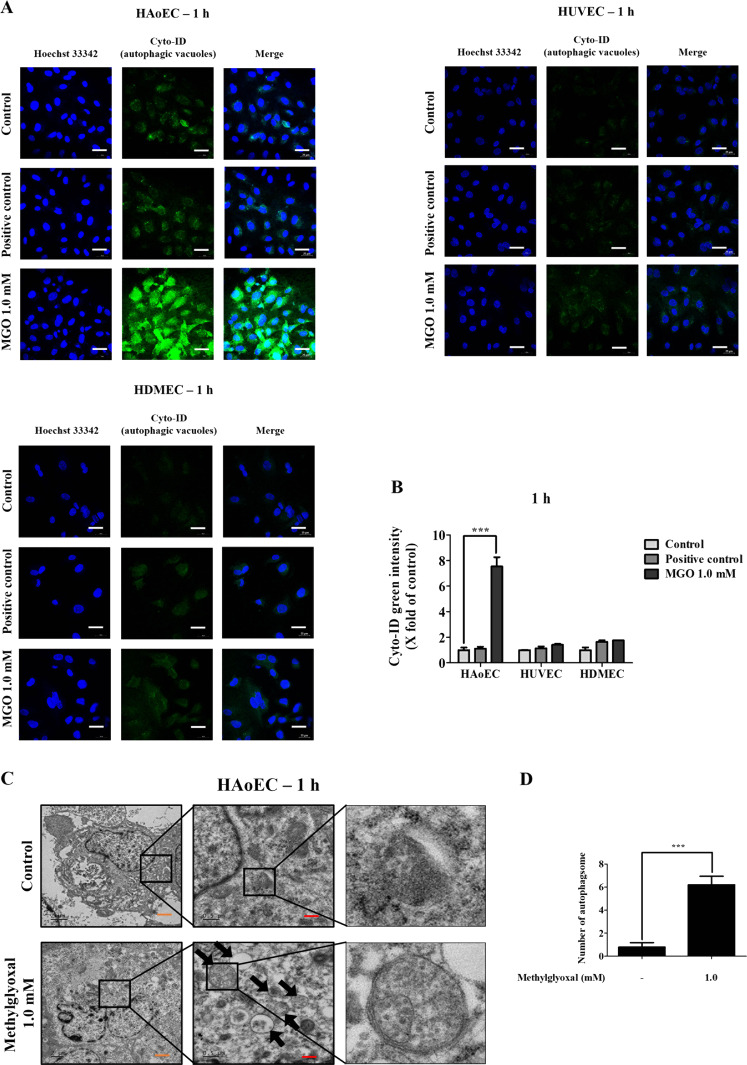
Effects of MGO-induced autophagic vacuoles in vascular endothelial cells. **a** HAoEC, HUVEC, and HDMEC were treated with a control or 1.0 mM of MGO for 1 h and were evaluated for autophagic induction by staining with a Cyto-ID® autophagy detection kit. Cells were treated with a mixture of chloroquine (10 μM) and rapamycin (0.5 μM) for 1 h to make a positive control and were evaluated as described in (**a**). The stained cells were analyzed by confocal microscopy (×60 magnification). **b** Quantitative measurements of Cyto-ID green intensity were calculated using NIS-Elements imaging software. Scale bar indicates 25 μm. **c** Bio-transmission electron microscopic images of HAoEC treated with or without MGO. Untreated cells (control), MGO-treated cells. Abundant typical double-layer membrane autophagosomes (black arrows) observed in HAoEC treated with MGO (1.0 mM) for 1 h. **d** The static results of autophagosomes were calculated random TEM images. Scale bar indicates 0.5 and 2 μm. All data are shown as means ± SEM. *N* = 3 (***p < 0.001 vs. Control).

Cyto-ID® autophagy detection kits and a confocal microscope were used to further confirm MGO-induced autophagy through measuring the autophagic vacuoles and by monitoring the autophagic flux in fixing cells. As shown in Fig. 2a, b, the fluorescence intensities of HAoEC, HUVEC, and HDMEC treated with MGO for 1 h were greater than those of the chloroquine (10 μM) and rapamycin (0.5 μM) positive controls, indicating MGO-induced autophagy in vascular EC and confirming the above-described results. The maximum increase in autophagic vacuoles and flux was found in HAoEC as compared to HUVEC and HDMEC (Fig. 2a, b) concluding MGO is more specific and more sensitive to HAoEC.

### MGO induces autophagosomes by bio-TEM in HAoEC

To identify changes in autophagosome formation, newly formed autophagosomes in HAoEC were evaluated using a bio-TEM. The number of autophagosomes (black arrow) was significantly increased in MGO-induced HAoEC compared to the untreated group (Fig. 2c). The statistic results revealed significant differences between control and MGO 1.0 mM treated groups (Fig. 2d).

### MGO induces cell viability or toxicity in vascular ECs

**Fig. 3:**
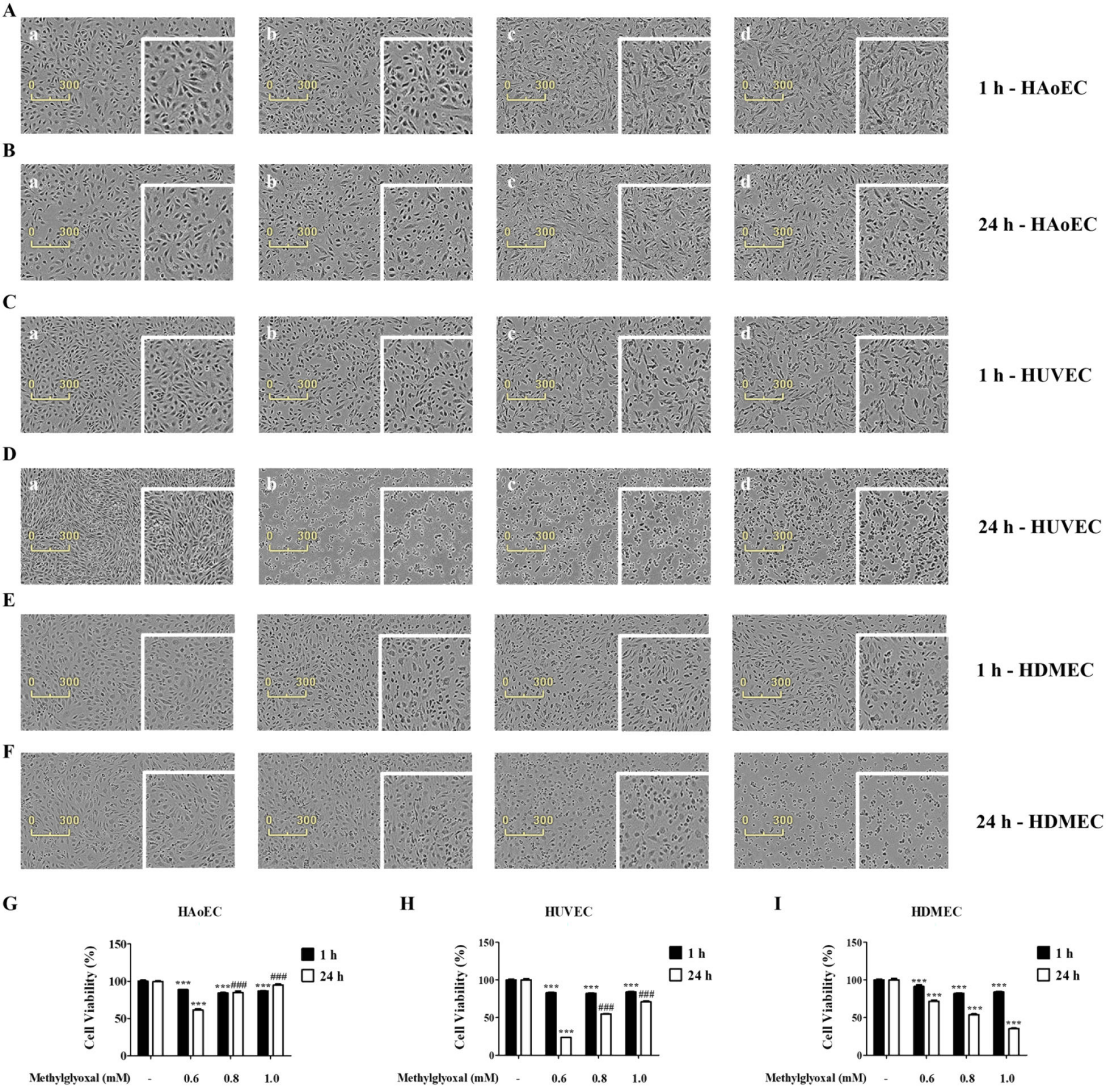
Effects of MGO on HUVEC and HAoEC viability. **a**–**f** Photomicrographs of HAoEC, HUVEC, and HDMEC: **a** Control; **b** MGO 0.6 mM; **c** MGO 0.8 mM; **d** MGO 1.0 mM for 1 and 24 h. **g**–**i** The viability of HAoEC, HUVEC, and HDMEC under treatment with three concentrations (0.6, 0.8, and 1.0 mM) of MGO for 1 and 24 h. Cell viability was measured using MTT assay, as detailed in the methods section. Scale bar indicates 300 μm. All data are shown as means ± SEM. *N* = 3 (***p < 0.001 vs. Control, ^###^*p* < 0.001 vs. MGO 0.6 mM).

To determine whether MGO was toxic to cells in any way, cell viability and morphological analyses were conducted after 1 and 24 h in HAoEC, HUVEC, and HDMEC (Fig. 2). In addition, the morphological changes to cells at different concentrations of MGO (0.6, 0.8, and 1.0 mM) were examined Fig. 3a–f. MGO (0.8, 1.0 mM) exerted few cytotoxicity effects on HAoEC and HUVEC when cells were treated for 1 and 24 h. However, cell viability decreased in a dose-dependent manner upon treatment MGO in HDMEC. It was observed that HAoEC viability was higher than HUVEC, HDMEC viability when cells were treated with MGO for 24 h (Fig. 3g–i).

### MGO induces cytoskeleton protein damage in vascular ECs

**Fig. 4:**
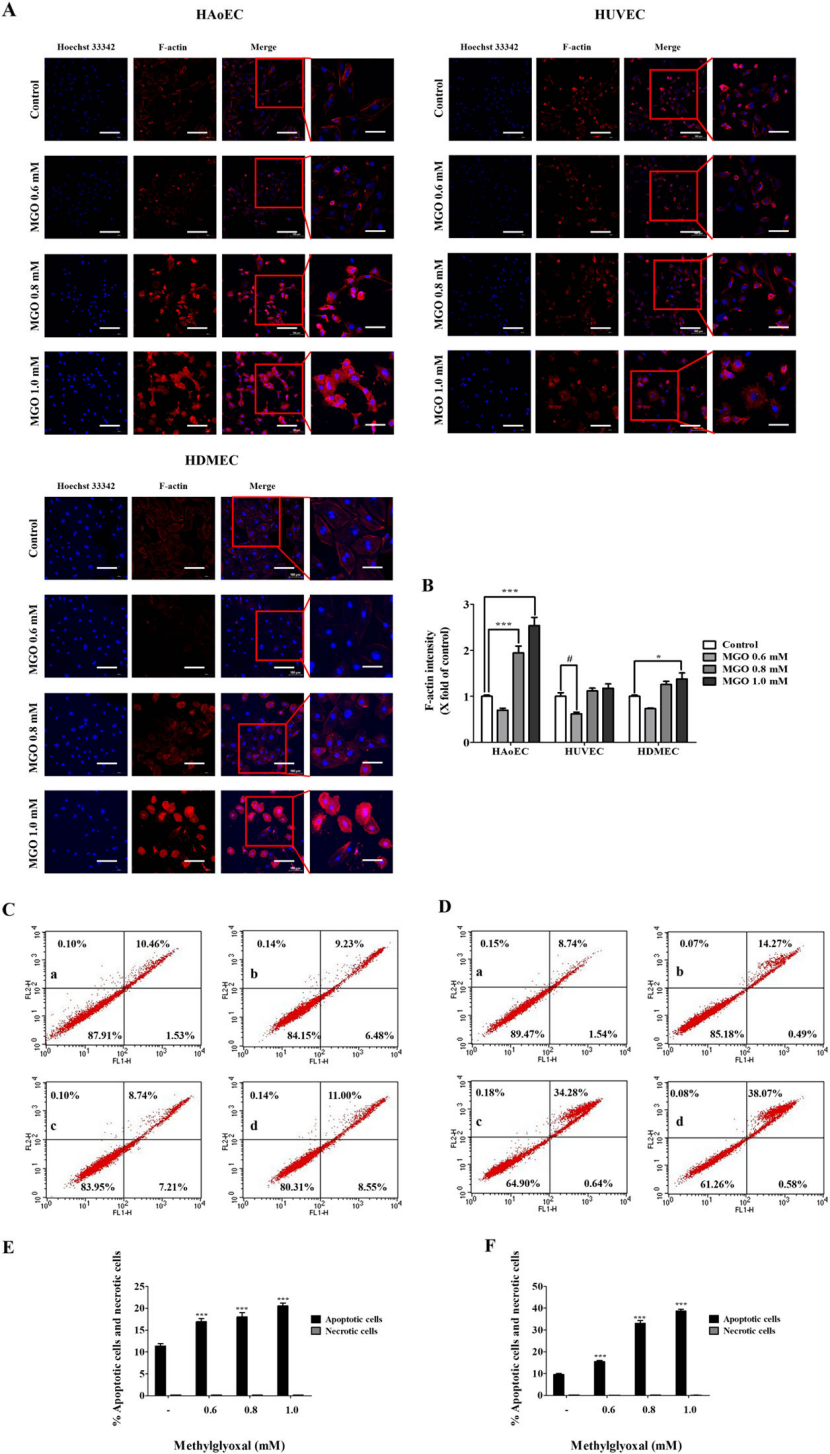
Effects of MGO-induced cytoskeletal proteins and apoptosis in vascular endothelial cells. **a** HAoEC, HUVEC, and HDMEC were treated with MGO for 1 h. Cells were stained with Hoechst 33342 (blue; nuclear) and F-actin (red) Alexa Fluor 555® Phalloidin, and measured using confocal microscopy. Scale bar indicates 100 μm. **b** Quantitative measurements of F-actin fluorescence intensity were determined using NIS-Elements imaging software. Scale bar indicates 100 μm. **c**, **d** Representative cytograms of Annexin V-FITC and PI staining of MGO-induced HAoEC. Cells were treated with several concentrations (0.6, 0.8, and 1.0 mM) of MGO for 1 and 24 h. After 1 and 24 h, the concentrations of viable (Annexin V-FITC and PI negative cells), early stage apoptotic (Annexin V-FITC positive, PI negative cells), late-stage apoptotic (Annexin V-FITC positive, PI-positive cells), and necrotic (PI-positive cells) cells were evaluated by flow cytometry. **a** Control; **b** 0.6 mM MGO; **c** 0.8 mM MGO; and **d** 1.0 mM MGO. **e**, **f** Analysis value of control, early stage apoptotic, late-stage apoptotic, and necrotic cells as evaluated by flow cytometry. All data are shown as means ± SEM. *N* = 3 (*p < 0.05, **p < 0.01, ***p < 0.001 vs. Control, ^#^*p* < 0.05, ^##^*p* < 0.01, ^###^*p* < 0.001 vs. MGO 0.6 mM).

HAoEC, HUVEC, and HDMEC were used to explore MGO-induced cytoskeleton protein damage using a confocal. Cells treated with several concentrations (0.6, 0.8, and 1.0 mM) of MGO for 24 h were stained with Hoechst 33342 and F-actin dye. The morphological changes to cells at several concentrations (0.8 and 1.0 mM) of MGO in HAoEC, HUVEC, and HDMEC were examined in Fig. 4a. Also, MGO (0.8 and 1.0 mM) exerted significantly increased F-actin intensity effect on HAoEC and HDMEC when cells were treated for 24 h (Fig. 4b). However, F-actin intensity significantly decreased upon treatment MGO 0.6 mM in HAoEC, HUVEC, and HDMEC.

### MGO induces apoptosis in HAoEC

HAoEC were used to explore MGO-induced apoptosis and necrosis using a flow cytometer. Cells treated with several concentrations (0.6, 0.8, and 1.0 mM) of MGO for 1 h and 24 h were stained with FITC Annexin V and PI dye. As shown in Fig. 4c–f, MGO significantly increased apoptosis production in a dose-dependent manner in HAoEC.

### MGO induces ROS production in HAoEC

**Fig. 5:**
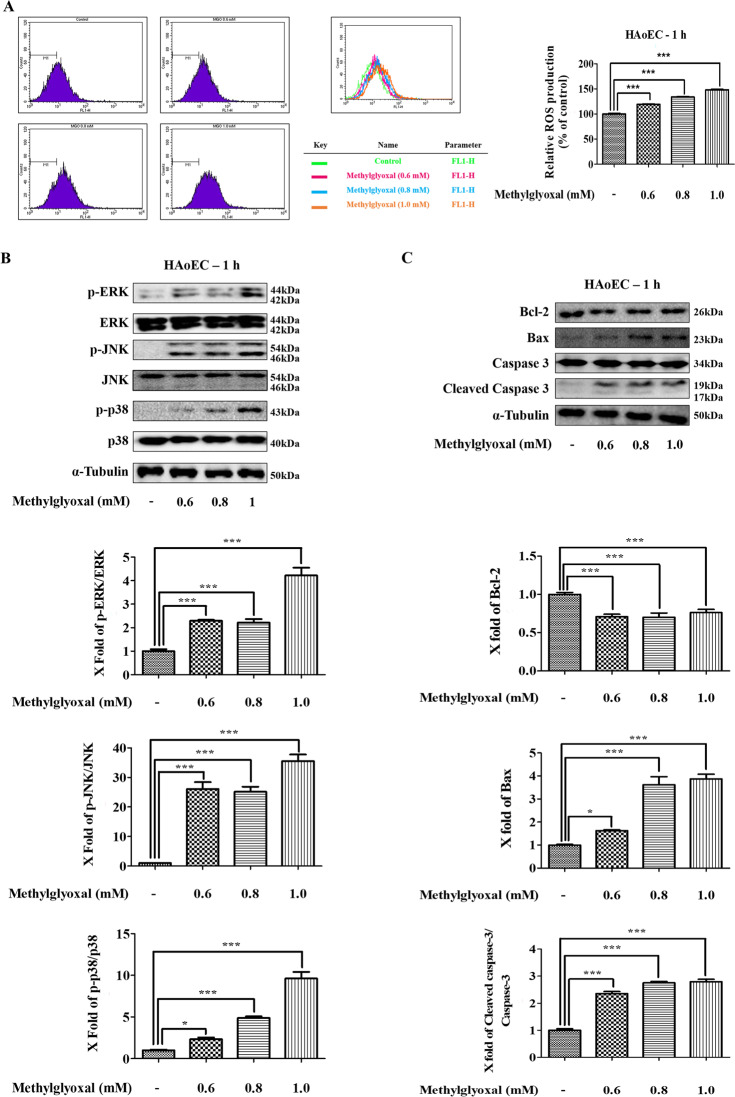
Effects of MGO on apoptosis-related proteins and MAPKs signaling pathway activation through ROS generation. The levels of ROS in MGO-treated HAoEC were measured after 1 h by flow cytometry with DCFH-DA dye. **a** The number of cells is designed against the dichlorofluorescein fluorescence detected by the FL-1 channel. Relative ROS production is represented in each histogram. **b**, **c** MGO-treated HAoEC were measured for the expression levels of the MAPKs signaling pathway, Bax, Bcl-2, caspase-3, cleaved caspase-3, and α-tubulin by western blots for 1 h. The protein levels of p-ERK, ERK, p-JNK, JNK, p-p38, p-38, Bax, Bcl-2, caspase-3, and cleaved caspase-3 were determined by the Image Lab analysis tool. All data are shown as means ± SEM. *N* = 3 (*p < 0.05, ***p < 0.001 vs. Control).

HAoEC were used to investigate MGO-induced ROS production using a flow cytometer. HAoEC treated with various concentrations (0.6, 0.8, and 1.0 mM) of MGO for 1 h were stained with DCF-DA dye, and changes in fluorescence intensity were observed by flow cytometer. As shown in Fig. 5a, MGO significantly increased ROS production in a dose-dependent manner in HAoEC. This result suggests that MGO induces ROS production in HAoEC.

### MGO induces MAPKs signaling pathways in HAoEC

MAPKs signaling pathways play important roles in regulating proliferation, apoptosis, autophagy, and cell cycle arrest^38^. Therefore, the protein levels of these factors were investigated by western blot analysis. Western blot analysis showed MGO-activated phosphorylation of ERK, JNK, and p38 in HAoEC (Fig. 5b). The band intensities increased significantly in a dose-dependent manner between phosphorylated and total-forms (Fig. 5b). Consequently, the autophagy marker known as the LC3-II/LC3-I ratio increased through the MAPKs signaling pathway.

### MGO induces Bax and caspase-3 expression and decreases Bcl-2 expression

To investigate the effects of MGO-induced apoptotic cell death, the expression levels of anti-apoptotic and pro-apoptotic proteins such as Bax, Bcl-2, and cleaved caspase-3 were examined. Among the pro-apoptotic proteins, Bax expression levels increased in a dose-dependent manner, whereas MGO decreased Bcl-2 expression levels compared to the control group (Fig. 5c). Apoptosis-related cleaved caspase-3, Bax, and Bcl-2 protein levels also increased in a dose-dependent manner (Fig. 5c). Therefore, both autophagy and apoptosis occur with MGO treatment in HAoEC.

### MGO inhibits cell proliferation, migration, and tube formation in vascular EC

**Fig. 6:**
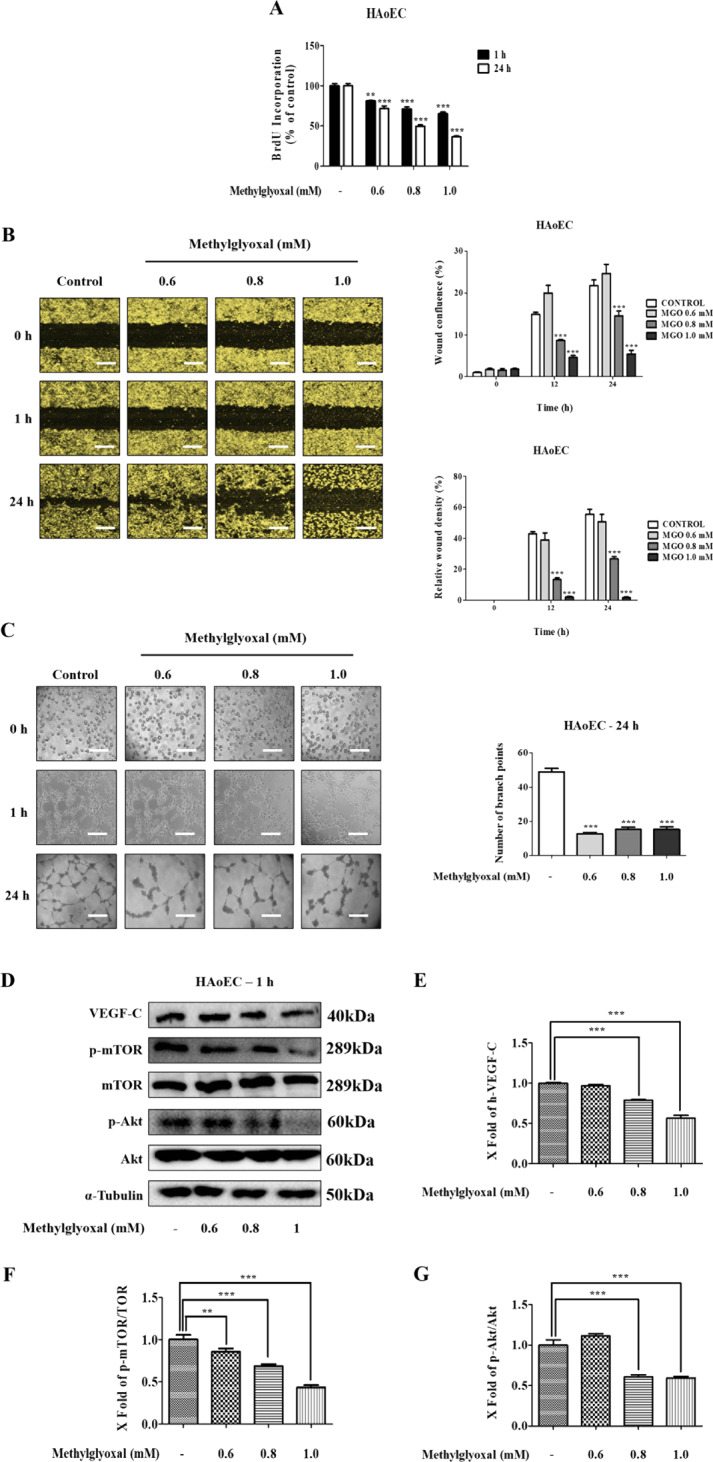
Effects of MGO on the cell proliferation, cell migration, tube formation, and PI3K/Akt/mTOR signaling pathway in HAoEC. Cells exposed to different concentrations (0.6, 0.8, and 1.0 mM) of MGO. **a** HAoEC in the BrdU assay proliferated lower when cultured with MGO compared with the control. **b** MGO-treated cells at 0 and 24 h subjected to scratch wound healing assays. Cell migration was quantified by wound confluence and relative wound density in each group. Scale bar indicates 300 μm. **c** The representative photomicrographs exhibiting the effects of MGO inhibition on the aortic-like morphology of HAoEC at 24 h after seeding onto Matrigel. Quantitative analyses of tube formation were carried out by counting the number of branches from three randomly selected fields per well. Scale bar indicates 100 μm. **d** MGO-treated HAoEC were evaluated for the expression levels of autophagy-associated proteins in the PI3K/Akt/mTOR signaling pathway. The protein expression levels of VEGF-C, Akt, p-Akt, p-mTOR, mTOR, and α-tubulin were investigated by western blots for 1 h. **e**–**g** The densitometry values of VEGF-C, p-Akt/Akt, and p-mTOR/mTOR were evaluated using the Image Lab analysis tool. All data are shown as means ± SEM. *N* = 3 (**p < 0.01, ***p < 0.001 vs. Control).

To investigate the role of MGO on the migration, tube formation, and proliferation of HAoEC, assays were performed. The cell migration assays revealed that cell proliferation rate significantly decreased in a dose-dependent manner compared to the untreated group (Fig. 6a). To evaluate the role of MGO in inhibiting the angiogenic response, cell migration assays were performed. The results, as shown in Fig. 6b determined that cells treated with MGO exhibited a lower migration rate (wound confluence and relative wound density) compared with the untreated control group. In addition, microscopic images taken after 24 h of treatment revealed that tube formation inhibition occurred in a dose-dependent manner (Fig. 6c).

### MGO inhibits the PI3K/Akt/mTOR signaling pathway in HAoEC

In order to understand the mechanisms of action of MGO-induced autophagy, the autophagy regulation-associated levels of Akt and mTOR proteins, which are involved in the early stage of autophagy, were investigated. A previous study has shown that the PI3K/Akt/mTOR signaling pathway plays a vital role in autophagy^39^. In addition, inhibition of the phosphorylation activities of Akt and mTOR is needed to promote autophagy^40^. Therefore, we investigated whether MGO-induced autophagy occurs via inhibition of the PI3K/Akt/mTOR signaling pathway. HAoEC were treated with several concentrations of MGO (0.6, 0.8, and 1.0 mM) for 1 h, and western blot analysis was carried out. Protein expression levels of VEGF-C, p-Akt/Akt, and p-mTOR/mTOR decreased in a dose-dependent manner (Fig. 6d–g). These results suggest that MGO induces autophagy in HAoEC through inhibition of the phosphorylation activities of Akt and mTOR via the PI3K/Akt/mTOR signaling pathway.

### The effect of Aminoguanidine (AG) on MGO-induced autophagy in HAoEC

**Fig. 7:**
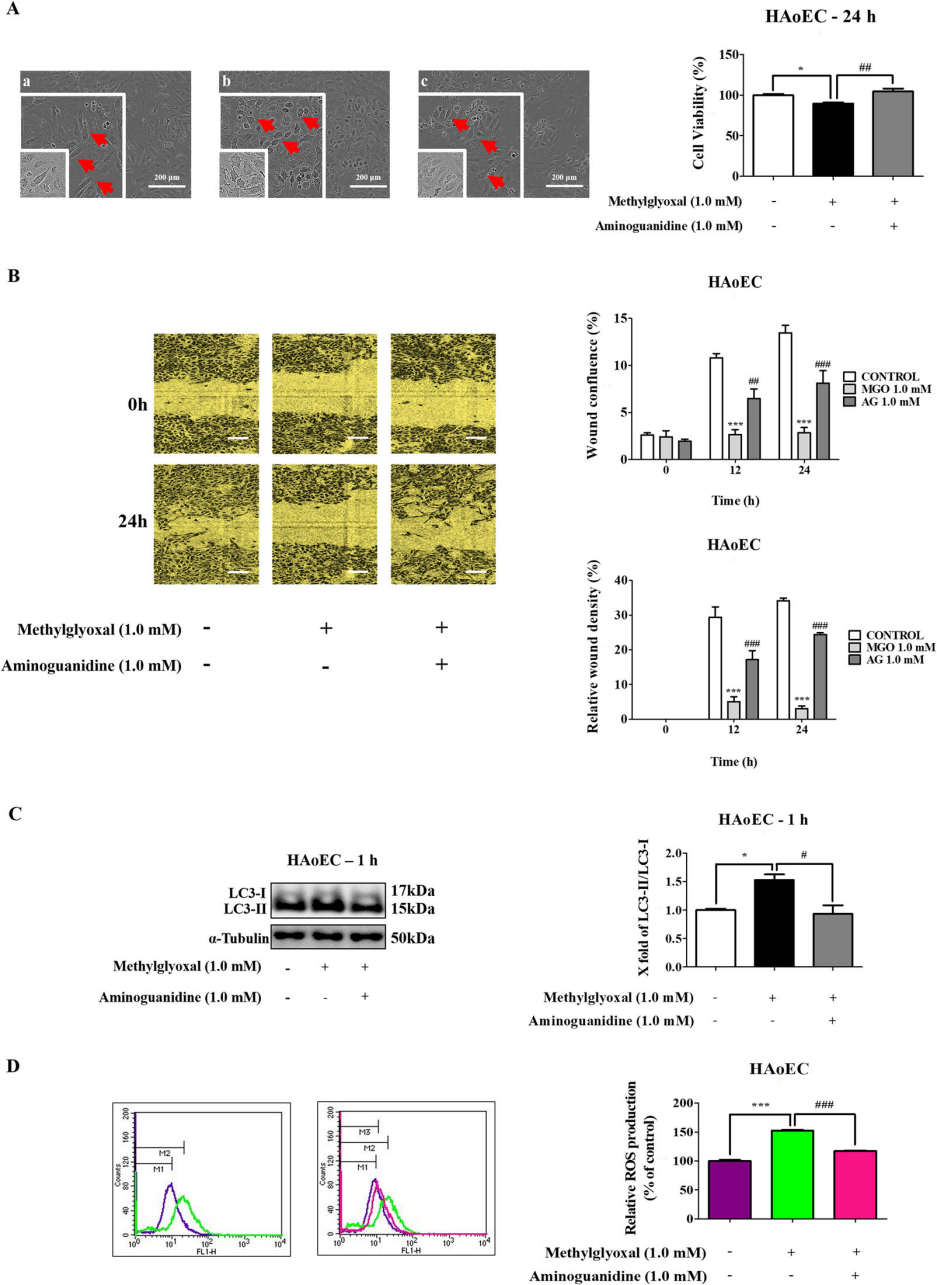
Effects of AG on MGO-induced autophagy in HAoEC. Cells exposed to MGO-treated HAoEC without (−) or with (+) AG. **a** Photomicrographs of HAoEC: **a** control; **b** MGO 1 mM; **c** MGO + AG (1.0 mM). Cell viability was measured using MTT assays, as detailed in the methods section. **b** HAoEC were subjected to scratch wound healing assays for 0 and 24 h. Cell migration activity was quantified by wound confluence and relative wound density in each group. **c** Cells were evaluated for the expression of the LC3-I and LC3-II autophagy-associated proteins. The protein expression levels of LC3-I, II, and α-tubulin were measured by western blots at 1 h. **d** HAoEC were pretreated with 1.0 mM AG for 1 h, followed by 1.0 mM MGO for 1 h. The levels of ROS generation from DCFH-DA dye was analyzed by flow cytometry. Quantitative measurements of fluorescent intensity were measured using a flow cytometry analysis system. Scale bar indicates 200 μm. All data are shown as means ± SEM. *N* = 3 (*p < 0.05, ***p < 0.001 vs. Control, ^#^*p* < 0.05, ^##^*p* < 0.01, ^###^*p* < 0.001 vs. MGO 1.0 mM).

Pretreatment with AG 1.0 mM changed cell shape (red arrows) in HAoEC (Fig. 7a). In addition, pretreatment lessened the effects of AG on MGO-induced cell migration by the wound healing system. The results, as shown in Fig. 7b determined that cells treated with AG exhibited a higher migration rate (wound confluence and relative wound density) compared to the untreated MGO group. Also, pretreatment with AG 1.0 mM for 1 h significantly decreased MGO-induced LC3-II/LC3-I (Fig. 7c). As shown in Fig. 7d, 1.0 mM AG decreased MGO-induced ROS generation in HAoEC.

## Discussion

Autophagy (self-ingestion) and apoptosis (self-killing) play important roles in cell development, differentiation, and repair^34,41^. Maintaining autophagy and apoptosis is important in maintaining cellular homeostasis. In previous studies, based on morphological data, the following three major types of programmed cell death have been reported: type I apoptotic cell death (apoptosis), type II autophagic cell death (autophagy), and type III necrotic cell death (necrosis)^42,43^. Chen et al.^41^ highlighted that the molecular mechanisms behind several types of cell death are distinct but also overlapping. Autophagy and apoptosis work together to maintain angiogenesis by interacting with different angiogenic factors^37^. Previously, it has been shown that MGO exhibits a protective role against injures by inducing autophagy in human brain microvascular EC^44^. However, until now, the role of MGO in increasing or decreasing autophagy and apoptosis to regulate angiogenesis in HAoEC had not been studied. Therefore, the current study was conducted to address this question, and the results were remarkable.

It has been studied and mentioned in previous research that microtubule-associated protein 1A/1B-light chain 3 (LC3) lipidation is necessary for autophagy, and that double-membrane structures are associated with the conversion of LC3-I to LC3-II^45^. The present study found that MGO increases this conversion rate and activates autophagy for 1 h in a dose-dependent manner (Fig. 1a, b), but that these activities decrease at 24 h post-treatment in HAoEC (Fig. 1c, d). This decreased level of LC3-II is associated with the co-degradation of autophagic cargo in lysosomes^46^. In terms of LC3 conversion, MGO induces more autophagy in HAoEC than in HUVEC and HDMEC (Fig. 1e, f). HAoEC, being a vital component of the body, may respond more sensitively to MGO treatment. Increased amounts of LC3-II may be associated with other metabolites including glucose and glyoxal but the results of the present study indicated that MGO-induced autophagy to a greater extent than other metabolites (Fig. S1).

The results of TEM, a typical autophagy detection method, indicated that the number of autophagosomes was significantly increased in MGO-treated cells (Fig. 2c, d). These results indicated that MGO activates autophagy in HAoEC. However, autophagy is a multistep and highly dynamic process. Accordingly, these results should be carefully considered, because an increased LC3-II/LC3-I ratio or formation of autophagosomes may reflect either the generation of autophagy or a decrease in autophagic turnover. In addition, increased LC3-II levels are related to increased autophagy flux and the presence of autophagic vacuoles during autophagy^28,47^. Thus, increased autophagic flux and the presence of autophagic vacuoles also suggest the induction of autophagy. The findings of the present study also showed an increased level of LC3-II in conjunction with increased autophagic flux and autophagic vacuoles in MGO-treated cells (Fig. 2a, b). Moreover, autophagic flux alterations have the potential to contribute to disease processes such as pulmonary hypertension and atherosclerosis^48,49^. There is growing evidence that autophagy has roles in cell proliferation, cell migration, and tube formation, and that increased levels of autophagy indirectly inhibit this cellular processes^4,50,51^. Our experimental results indicated that MGO induced the anti-angiogenesis via inhibiting the migration, cell proliferation, and tube formation (Fig. 6a-c, S3) in HAoEC, HUVEC, and HDMEC. In HAoEC, MGO was meore effective in inhibiting prolifreration and tube formation in comparison with HUVEC and HDMEC. Additionally, cytoskeleton proteins directly contribute to migration during angiogenesis, a process throughout which cytoskeletal remodeling occurs in EC and directly affects cellular morphology and movement^52–54^. One of the cytoskeleton proteins, F-actin, is considered as a biomarker of autophagy like LC3 and its expression has been noted^55,56^. The results showed the activation of F-actin fluorescence intensity and cellular morphology transformation which indicates the autophagy induction and cellular damage on MGO treatment. (Fig. 4a, b).

Scherz-Shouval et al.^57^ reported that ROS stimulates autophagy, protects cells from starvation by activating autophagy-related proteins, and improves vascular functions. As previously reported that increased ROS levels inhibit the PI3K/AKT/mTOR signaling pathway and induce MAPKs signaling pathway^58^ through effecting their related proteins. The present results also demonstrated the inhibition of p-AKT and p-mTOR, which are involved in the PI3K/AKT/mTOR signaling pathway, and the induction of p-JNK, p-p38, and p-ERK, which are involved in the MAPKs signaling pathway (Figs. 5b and 6d–g) Furthermore, P13K/AKT/mTOR, VEGF-C, Bcl-2 families, ROS, p53 are considered as major molecules to be involved in angiogenesis-related autophagy/apoptosis mechanism to activate disease conditions in a variety of cells^41,59,60^. Upon investigation with MGO treatment, we have found decreased expression of p-Akt, p-mTOR, and VEGF-C, another linked signaling pathway of MGO-induced autophagy to inhibit angiogenesis. Therefore, we investigated that the relations among these three types of cell death and explores the significance of cell death under the specific conditions of human diseases, particularly diabetes and diabetic complications such as ED.

AG, an effective inhibitor of AGEs formation, prevents diabetic-related vascular complications through ameliorating MGO-induced glucotoxicity, apoptosis, and oxidative stress in ECs^28,29^. Therefore, the effects of AG on MGO-induced HAoEC autophagy were examined in vitro. The findings suggested that AG prevents MGO-induced HAoEC autophagy through changing cellular morphology, increasing cell migration, and decreasing the LC3-II/LC-I ratio and ROS generation (Fig. 7).

## Conclusion

**Fig. 8:**
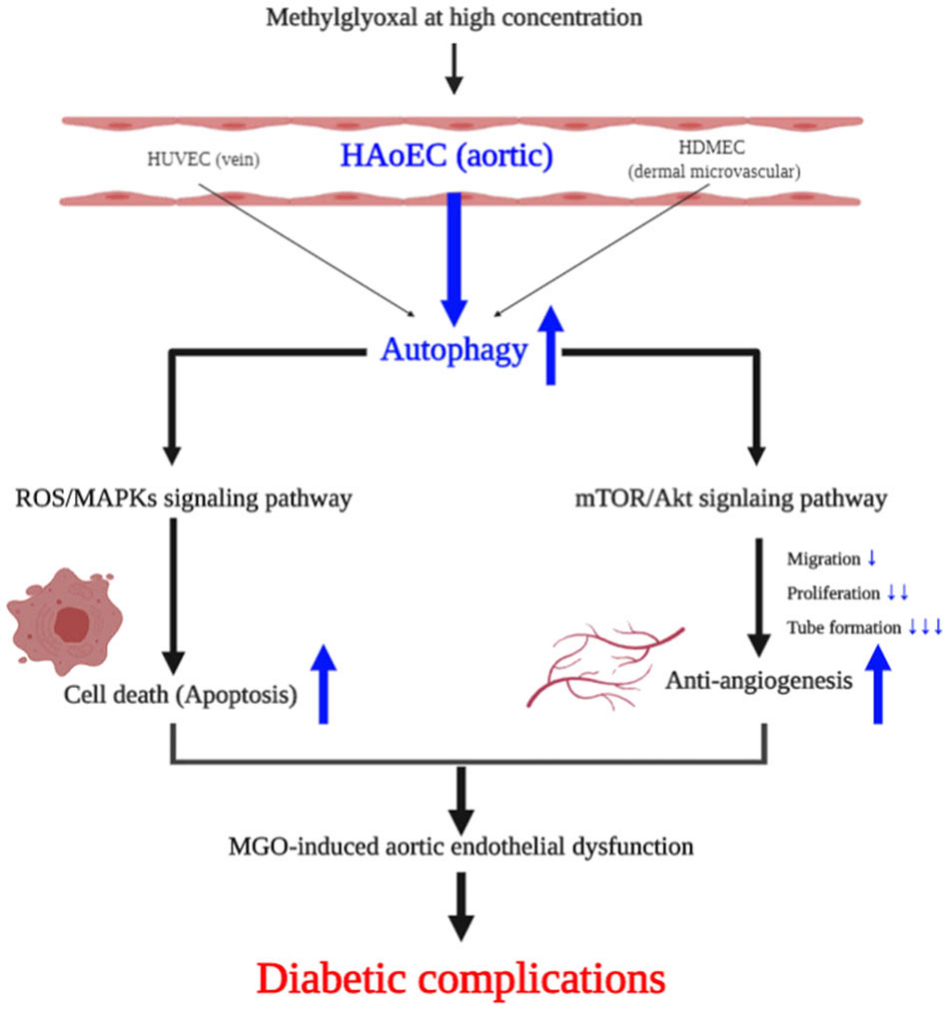
Possible molecular mechanisms of MGO-induced aortic endothelial dysfunction in human vascular ECs. This schematic description of triggers in autophagy upon MGO treatment. Blue and black arrow define the activity of MGO.

This study demonstrated that MGO induces aortic ED via autophagic cell death and anti-angiogenesis in HAoEC. Specifically, it proves that MGO activates the ROS-mediated MAPKs signaling pathway, autophagy/apoptosis and inhibits the Akt/mTOR signaling pathway, cellular migration, proliferation, and tube formation via the anti-angiogenesis process (Fig. 8). It has been proposed that MGO plays a vital role in vascular damage to EC and in the development of vascular disease. Thus, the ability of therapeutic agents to prevent MGO-induced dysfunction in EC may be effective against the development of diabetic complications, and the underlying signaling network may be a new therapeutic target. Based on these new findings, in the future, new therapeutic agents can be developed that focus on preventing and treating diabetic vascular complications in clinical practice.

## Supplementary information

Supplementary Figure Legends

Fig. S1 Effects of glucose and glyoxal on expression levels of autophagy-related proteins LC3-I and LC3-II in HAoEC.

Fig. S2 Effects of MGO-induced autophagic vacuoles in vascular endothelial cells.

Fig. S3 Effects of MGO on the proliferation, migration, and tube formation in HUVEC and HDMEC.

Fig. S4 Effects of MGO-induced autophagy-related protein expression in HAoEC.

Fig. S5 Effects of NAC, Quercetin, and AG on MGO-induced ROS generation in HAoEC.

Fig. S6 Effects of U0126, SP 600125, SB 203580 on autophagy through MAPKs signaling pathways in HAoEC.
