# Supplementary material for: Molecular mechanisms of methylglyoxal-induced aortic endothelial dysfunction in human vascular endothelial cells
Source: Cell Death Dis. 2020 May 28;11(5):403. doi: 10.1038/s41419-020-2602-1 (PMC7256048; doi:10.1038/s41419-020-2602-1)
Supplement: Supplementary file 1 — Supplementary Figure Legends [file 41419_2020_2602_MOESM1_ESM.docx]

**Supplementary Figures Legends**

**Fig. S1 Effects of glucose and glyoxal on expression levels of autophagy-related proteins LC3-I and LC3-II in HAoEC.** (A) The structure of a; glucose, b; methylglyoxal, c; glyoxal. (B), (C) Glucose and glyoxal-treated HAoEC were examined for the expressions of autophagy-associated proteins, LC3-I and LC3-II. Cells were treated with three concentrations (0.6, 0.8, and 1.0 mM) of glucose and glyoxal. The protein expression levels of LC3-I, LC3-II, and α-tubulin are shown after treatment for 1 h. (D), (E) The densitometry levels of LC3-I and LC3-II were evaluated by the Image Lab analysis tool. All data are shown as mean ± SEM. N = 3 (**p < 0.01, ***p < 0.001 vs. Control)

**Fig. S2 Effects of MGO-induced autophagic vacuoles in vascular endothelial cells.** (A) HAoEC, HUVEC, and HDMEC were treated with a control or several concentrations (0.6, 0.8 mM) of MGO for 1 h and were measured for autophagic induction via staining with a Cyto-ID® autophagy detection kit. Cells were treated with mixture chloroquine (10 μM) and rapamycin (0.5 μM) for 1 h to make a positive control and were evaluated as described in (A). (B) Quantitative measurements of Cyto-ID green intensity fluorescence intensity were determined using NIS-Elements imaging software. Scale bar indicates 25 μm. All data are shown as mean ± SEM. N = 3 (**p < 0.01 vs. Control)

**Fig. S3 Effects of MGO on the proliferation, migration, and tube formation in HUVEC and HDMEC.** Cells were exposed to different concentrations (0.6, 0.8, and 1.0 mM) of MGO (A) BrdU assay revealed a lower proliferation of HUVEC and HDMEC when cultured with MGO in comparison with the control. (B) Cells were treated with MGO for 0 h and 24 h and exposed to scratch wound healing assay. Cell migration activity was evaluated by wound confluence and relative wound density in each group. (C) The representative photomicrographs exhibiting the effects of MGO inhibition on the vein-like morphology of HUVEC and HDMEC at 24 h after seeding onto matrigel. Quantitative analysis of tube formation was conducted by counting the number of branches from three randomly selected fields per well. Scale bar indicates 300 μm. All data are shown as mean ± SEM. N = 3 (*p < 0.05, **p < 0.01, ***p < 0.001 vs. Control)

**Fig. S4 Effects of MGO-induced autophagy-related protein expression in HAoEC.** (A) MGO-treated HAoEC were evaluated for the expression levels of the LC3-I, LC3-II, p62, Beclin-1, and α-tubulin by western blots for 1 h. (B-D) The protein levels of LC3-I, LC3-II, p62, Beclin-1, and α-tubulin were determined by the Image Lab analysis tool. All data are shown as mean ± SEM. N = 3 (*p < 0.05, **p < 0.01, ***p < 0.001 vs. Control)

**Fig. S5 Effects of NAC, Quercetin, and AG on MGO-induced ROS generation in HAoEC.** (A, B) HAoEC were pretreated with NAC, Quercetin, and AG for 1 h, followed via 1.0 mM MGO for 1 h. The levels of ROS generation in HAoEC were evaluated against the DCFH-DA detected by flow cytometry. Relative ROS generation is represented in each histogram. (C) Quantitative measurements of fluorescent intensity were calculated using BD CellQuest^TM^ pro software. All data are shown as mean ± SEM. N = 3 (***p < 0.001 vs. Control, ##p < 0.01, ###p < 0.001 vs. MGO 1.0 mM)

**Fig. S6 Effects of U0126, SP 600125, SB 203580 on autophagy through MAPKs signaling pathways in HAoEC.** (A, B) Representative western blots of LC3-I, II, total, and phosphorylated forms of MAPKs signaling pathway by MGO-induced HAoEC. Cells were pretreated with U0126 (p-ERK inhibitor), SP 600125 (p-JNK inhibitor), and SB 203580 (p-p38) for 1 h, followed by 1.0 mM MGO for 1 h. (C, D) The protein levels of LC3-I, LC3-II, p-ERK, ERK, p-JNK, JNK, p-p38, and p-38 were determined by the Image Lab analysis tool. All data are shown as means ± SEM. N = 3 (***p < 0.001 vs. Control, ###p < 0.001 vs. MGO 1.0 mM).
